# Making a Lecture Stick: the Effect of Spaced Instruction on Knowledge Retention in Medical Education

**DOI:** 10.1007/s40670-020-00995-0

**Published:** 2020-06-09

**Authors:** Marnix C. J. Timmer, Paul Steendijk, Sandra M. Arend, Marjolein Versteeg

**Affiliations:** 1 Center for Innovation in Medical Education, Leiden University Medical Center, Leiden, The Netherlands; 2 Department of Cardiology, Leiden University Medical Center, 2333 ZA Leiden, The Netherlands; 3 Department of Infectious Diseases, Leiden University Medical Center, Leiden, The Netherlands

**Keywords:** Educational neuroscience, Instructional design, Medical education, Spaced learning, Spacing effect

## Abstract

**Introduction:**

Poor knowledge retention is a persistent problem among medical students. This challenging issue may be addressed by optimizing frequently used instructional designs, such as lectures. Guided by neuroscientific literature, we designed a spaced learning lecture in which the educator repeats the to-be-learned information using short temporal intervals. We investigated if this modified instructional design could enhance students’ retention.

**Materials and Methods:**

Second-year medical students (*n* = 148) were randomly allocated to either the spaced lecture or the traditional lecture. The spaced lecture consisted of three 15-min instructional periods, separated by 5-min intervals. A short summary of the preceding information was provided after each interval. The traditional lecture encompassed the same information including the summary in the massed format, thus without the intervals. All students performed a baseline knowledge test 2 weeks prior to the lectures and students’ knowledge retention was assessed 8 days after the lectures.

**Results:**

The average score on the retention test (*α* = 0.74) was not significantly different between the spaced lecture group (33.8% ± 13.6%) and the traditional lecture group (31.8% ± 12.9%) after controlling for students’ baseline-test performance (*F*(1,104) = 0.566, *p* = 0.458). Students’ narrative comments showed that the spaced lecture format was well-received and subjectively benefitted their attention-span and cognitive engagement.

**Discussion and Conclusion:**

We were unable to show increased knowledge retention after the spaced lecture compared with the traditional lecture. Based on these findings, we provide recommendations for further research. Ultimately, we aim for optimized spaced learning designs to facilitate learning in the medical curriculum and to help educate health professionals with a solid knowledge base.

## Introduction

Medical students have a hard time recalling knowledge they acquired during medical training [1–4]. Since successful clinical reasoning is built upon a solid foundation of knowledge, medical education is facing a serious problem [5]. The issue of forgetfulness may partly result from currently used teaching practices. For instance, lectures are still widely used as a teaching modality, whereas it has been shown that traditional lecturing is less effective for retaining knowledge compared with active learning modalities [6, 7]. Besides implementing more active learning strategies, which is often time-consuming, educators may consider adjusting their current lecture practice. A strategy by which lectures may be optimized for enhancing knowledge retention is by implementing spaced learning: repeating knowledge or skills that have to be acquired in several learning sessions that are distributed over time. Spaced learning is usually contrasted with massed learning where information is packed together in a single learning session and only repeated consecutively, if repeated at all. In general, the current lecture practice can be considered massed learning. Spaced learning could be of value to instructional designs in medical education.

The effectiveness of spaced learning, i.e., the spacing effect, is based on evidence derived from a century of psychological research (for a meta-analysis, see Cepeda et al, 2006 [8] or Carpenter et al, 2012 [9]). The beneficial effects of spaced learning on retention have been shown for a variety of learning tasks concerning factual knowledge [10], conceptual knowledge [11, 12], and procedural knowledge [13].

Over the last 10 years, research has proven spaced learning to be successful in various medical disciplines, including surgery, urology, radiology, and general clinical reasoning [14–21]. Although spaced learning in medical education has been mostly investigated in online learning and simulation settings, there is a growing interest for spacing instructional designs as well. For instance, a study has shown that the dispersion of 4 h of direct instruction over 4 weeks, i.e., 1 h/week, significantly enhanced knowledge retention after 1 month [22]. Interestingly, neuroscientific research on mechanisms of memory implies that the spacing effect may already occur using much shorter intervals in the timescale of minutes to hours (for a review, see Smolen et al., 2016 [23]). This notion gave rise to our idea of implementing spaces within traditional massed 45–60-min lectures to promote long-term knowledge storage among medical students.

Researchers in higher education have already reported the successful application of spaces with short intervals. Kelley and Whatson [24] compared a 4-month biology course with a single 60-min spaced learning session. Students following the spaced learning session repeatedly received an intensive 20-min presentation (three times in total), intervened by 10-min breaks. Students’ final test results showed that repetition combined with these relatively short spaces can establish long-term memory. Recent initiatives were inspired by these findings and have illustrated the benefits of spaced instruction in different educational contexts [25, 26].

These educational initiatives promoting the use of short intervals to enhance long-term memory formation are inspired by neuroscientific evidence regarding the mechanisms of memory. An important phase in the process of long-term memory formation is the stabilization of a memory trace after the initial acquisition, referred to as *consolidation* [27]. Research has shown that consolidation of memory on the molecular level, referred to as long-term potentiation (LTP), is elicited particularly by spaced trials and to a lesser extent by massed trials [28, 29]. Importantly, memory consolidation involves various molecular processes that each have their own temporal dynamics. Some of these processes occur on the timescale of seconds to minutes and may contribute to the superiority of spaced learning [30–37] and the use of short spaces in particular.

Based on previous educational experiments in higher education and the evidence derived from neuroscience, we aimed to examine the effect of short spaces on knowledge retention in medical students. Therefore, we compared a spaced lecture design with a traditional massed lecture and measured students’ knowledge retention. We believe that the potential benefits of incorporating short spaces during teaching might help medical educators to make their lectures more effective.

## Materials and Methods

### Participants and Setting

Second-year medical students enrolled in a course on disease mechanisms at the Leiden University Medical Center (LUMC) were invited to voluntarily participate in the study. More than 80% of contact hours in this course consist of lectures. The intervention was conducted in a lecture on the Dutch national vaccination program. In previous academic years, information about the national vaccination program was covered by a self-study assignment. This topic was selected for this spaced learning study specifically, because students had received no prior formal education on this topic. The lectures were delivered as live presentations in a lecture hall, supported by a digital slideshow (Microsoft Powerpoint). This is common practice for lecturing at the LUMC.

### Ethical Considerations

Study participation was on a voluntary basis as the lectures were not mandatory. Students were notified that the supplied information was part of their exam material. Those who decided not to attend any session could still access the exam material using the existing self-study assignment. Students autonomously decided if their test results could be used for research purposes by signing the informed consent form prior to the baseline test, and again prior to the retention test. They were informed that data would be anonymized and that they could withdraw their consent at any given time. Moreover, they were ensured that the test results would not affect their course grades. Students did not receive any additional credit for their participation. The study protocol was reviewed and approved by the Educational Research Review Board of the LUMC: OEC/ERRB/20180612/2.

### Study Design

**Fig. 1:**
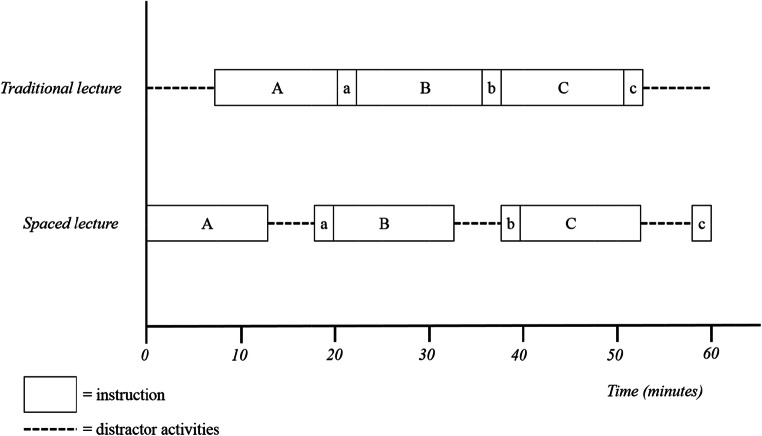
Illustration of the design of the control lecture (traditional) and the experimental lecture (spaced). The capital letters (A, B, and C) represent the regular instructional phase on the specific topics. The small letters (a, b, and c) represent short small summaries of the previous instruction block. In the experimental group, the regular instructional phase and small summaries were intervened by a 5-min gap, where students were asked to perform an origami task. The traditional lecture was preceded and followed by an origami task, but the instructional phase lasted 45 consecutive minutes. Both lecture sessions ended after 60 min

This was an experimental study for which two lectures were designed: an experimental lecture based on spaced learning principles, i.e., spaced lecture, and a control lecture using the traditional, massed approach, i.e., traditional lecture. Participants were randomly allocated to one of the lecture sessions. The spaced and traditional lectures were held consecutively on one day to facilitate that both lectures could be given by the same lecturer (SMA). The lectures were video recorded, to explore and reveal any substantial differences if suspected by the test results. The lecturer was a highly experienced teacher and is considered an expert in the field of infectious diseases. To assess students’ knowledge at baseline, they were tested approximately 2 weeks prior to the intervention, i.e., baseline test. The retention test was taken 8 days after the intervention. For a detailed scheme of the study procedure, see Fig. 1.

### Intervention

The lectures were designed for this experiment specifically, serving as a substitute for the self-study assignment that was used during preceding academic years. The lecture material comprised characteristics of the diseases covered by the Dutch national vaccination program (topic A), type and moment of vaccinations (topic B), and a regional and international comparison of participation and program components (topic C). Both lectures used the same supporting slides in identical order.

#### Spaced lecture

The total presentation time of 45 min was divided over three instructional periods of approximately 15 min, separated by breaks, i.e., intervening gaps, of 5 min, resulting in a 60-min lecture. To include repetition as a second key element in spaced learning, each break was followed by a short rehearsal of the previously presented information. To accomplish this, the lecturer used 2–3 summary slides covering the essentials (Fig. 1). We explicitly chose for summary slides as a passive rehearsal strategy, to be able for the lecturer to continue direct instruction after each break. In this way, we could study the effect of spacing on knowledge retention specifically, instead of inducing additional effects caused by active retrieval. The rationale for the 5-min spaces was based on our interpretation of neuroscientific literature, similar educational implementation studies, and practical feasibility [23, 24, 36, 37]. Topics A, B, and C were allocated to instructional periods 1, 2, and 3 respectively. During the 5-min breaks, students performed distractor activities (in our case, three different origami tasks) that were not in any way related to information provided in the lecture. Kelley and Whatson [24] also incorporated physical distractor activities into their design, as neuroscientists suppose this prevents cognitive interference with the memory formation process.

#### Traditional Lecture

The traditional lecture followed the conventional setup for lectures in medical education at the LUMC, which is a 45-min presentation without breaks. To control for potential confounders and ascertain the same time on task, the traditional lecture also contained the summary slides, i.e., repetition, and the same amount of time dedicated to distractor activities (Fig. 1). The latter were scheduled before and after the “massed” instructional session.

### Baseline Test and Retention Test

The baseline test and retention test consisted of short open-ended questions. The test questions were designed by the researchers and evaluated by two independent test experts. The lecturer was not involved in designing these tests and was not allowed to view the questions during the experiment. The tests were validated by performing a pilot test with independent associates (three PhD candidates with a medical degree and two undergraduate medical students), which reassured a score of 100% could be obtained by deriving the correct answers from the lecture slides.

Participants were tested for recall of factual information that was covered by the lecture. The test consisted of short open-ended questions to reassure that recall was assessed rather than recognition. Students could obtain one point for each correct answer. If students were asked to mention two or more aspects or items in their answers to a specific question, e.g., “Which two human papilloma virus (HPV) serotypes are primarily targeted by the HPV-vaccination?” they were only awarded the point if their answer was completely correct. No penalty was given for incorrect answers. Using a pre-made answer key, students’ answers were scored by one of the investigators, who was blinded for the lecture condition. All answers considered eligible for discussion were discussed by two researchers until consensus was reached.

#### Baseline Test

The baseline test was used to assess baseline knowledge regarding the topics covered in the lecture. The test consisted of 10 short open-ended questions. The test was performed in a lecture hall 2 weeks prior to the intervention to minimize priming effects.

#### Retention Test

Eight days after the intervention, students were invited to perform a retention test. This time frame was chosen because early research on memory indicates that forgetting declines exponentially and most of the forgetting occurs in the first week [38, 39]. Students were requested not to study the lecture material between the lectures and the retention test. The test included 30 short open-ended questions: the 10 questions of the baseline test plus 20 new questions. Several additional questions were included to reveal students who had violated the study-protocol, e.g., “Did you study the lecture material between lecture and the retention-test? If yes, in what way?”

#### Data Collection

All attendants at the baseline test were linked to an anonymous study code. This study code was used to couple retention test and baseline test data. If students admitted they had studied or did not follow their allocated lecture, i.e., violated the study protocol, they were marked and excluded from further analyses.

### Outcome Measures

The primary outcome measure was students’ performance on the full retention test. Final scores were expressed as the percentage of the maximal score. Secondary outcome measures were (i) performance on the 20 new retention test questions and on the 10 baseline test questions included in the retention-test, (ii) narrative comments of students and the lecturer on the used lecture formats. Test and item analyses were conducted for assessment of internal consistency and item characteristics (see Table 3 in the Appendix).

Narrative comments from the students and from the lecturer were gathered for qualitative assessment. To this end, students were actively encountered by the researchers immediately following the lectures and the retention test, and they were encouraged to express their thoughts on the lecture format in an informal way. At that time, students were unaware whether they were part of the intervention or control group. Narrative comments for both sessions were noted and stored digitally afterwards. The lecturer was interviewed after both lectures were concluded.

### Statistical Analyses

For the power analysis, the researchers agreed that a mean difference of at least one standard deviation should be detected, as this was regarded relevant for practice. Consequently, a minimum of 42 students (21 in each group) was needed to achieve 90% power at two-sided 5% significance.

The average total scores on the retention test and the subscores for the 20 new questions and for the 10 repeated baseline test questions were compared between study groups using ANCOVA tests, adjusting for students’ baseline knowledge scores. Reliability and item characteristics (difficulty and distinctiveness) of the retention test were evaluated by Cronbach’s alpha, *p* values, and Rir values (see Table 3 in the Appendix) [40]. Only data of students who completed both the baseline and the retention test were included for analysis. Those that did not follow their allocated lecture or restudied lecture material between the lecture and retention test violated the study protocol and consequently were marked and excluded from the analyses. Sensitivity analyses were carried out to reveal any major influence of these students on the outcome measures. If any of the test questions should be removed for any cause, another sensitivity analysis for its effect on score differences would be carried out.

## Results

**Fig. 2:**
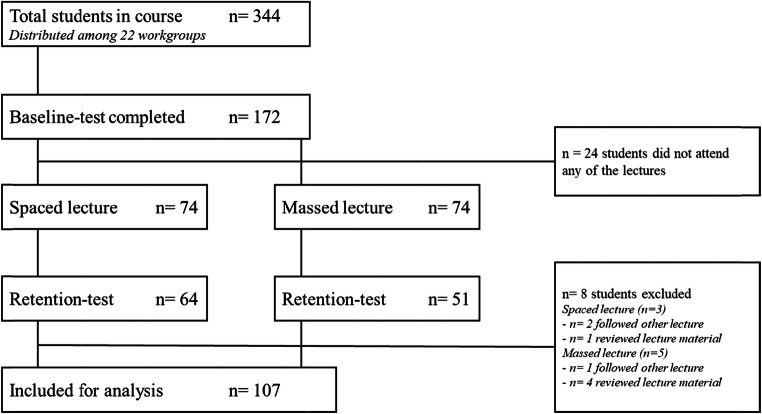
Participant flow diagram. Baseline test took place 14 days prior to the lecture. Retention test followed 8 days after the lecture

A total of 344 students were enrolled in the course, of whom 172 students completed the baseline test at the start of the course (Fig. 2). Halfway through the course, 148 of these students attended the lectures. Half of the participating students followed the spaced lecture (*n* = 74) and the other half followed the traditional lecture (*n* = 74). One week after the lectures, 115 students completed the retention test. Eight participants violated the study protocol, resulting in data of 107 students to be included for final analyses. On the retention test, there was a higher participation rate among students who attended the spaced lecture (86.5%) compared with the traditional lecture (68.9%) attendees (*χ2* (1, 148) = 6.5908, *p* = 0.010). However, further statistical analyses did not need to be adjusted since groups showed similar variance for all outcome measures.

### Demographics

The mean age of participants was 19.3 ± 0.9 years (Table 1). Of these participants, 90 (77.6%) were women, which resembles the overall gender distribution in the LUMC medical school. Both groups had similar average scores on the baseline test (spaced, 10.8% ± 8.8%; traditional, 10.4% ± 8.8%).

**Table 1:** Distribution across study groups. All are mean values unless stated otherwise

Variable	Spaced lecture (*n* = 61)	Traditional lecture (*n* = 46)
Women, proportion	68.9%	89.1%
Age, years (SD)	19.3 (0.9)	19.3 (0.9)
Pretest performance (SD)^1^	10.8% (8.8%)	10.4% (10.1%)

^1^Maximum score was 90%, after exclusion of question 9

### Retention

The average scores on the retention test were not significantly different between the groups (spaced, 33.8% ± 13.6%; traditional, 31.9% ± 12.9%, *F*(1,104) = 0.566, *p* = 0.454), also see Table 2. Separating the performance outcomes on the repeated original baseline test items from the novel items revealed no significant differences between groups (Table 2).

**Table 2:** ANCOVA test results for comparison of spaced lecture and traditional lecture cohorts on retention test performance

				ANCOVA test results		
	Spaced lecture, M (SD)	Traditional lecture, M (SD)	Difference (95% CI)	*F* value	*p* value	Effect size^1^
Retention test score (%)	33.8 (13.6)	31.8 (12.9)	2.0 (− 3.1 ; 7.2)	0.566	0.454	0.005
Retention test score on 20 new questions (%)	36.6 (14.9)	33.6 (13.2)	3.0 (− 2.5 ; 8.5)	1.108	0.295	0.011
Retention test score on 9 baseline test questions (%)	27.7 (14.7)	27.8 (15.7)	− 0.1 (− 6.0 ; 5.8)	0.007	0.934	0.000

*Notes.* For all ANCOVA tests, it applied that degrees of freedom 1 (df1) = 1 and degrees of freedom 2 (df2) = 104

*CI*, confidence interval

^1^Partial η2 effect size as predicted by the ANCOVA model

On one question of the retention test, the spaced lecture group very markedly outperformed the traditional lecture group (spaced, 59.0% correct; traditional, 2.2% correct). This extreme difference likely resulted from extra information that was unintentionally provided by the lecturer during the spaced lecture. Consequently, we excluded this question in our analyses which resulted in a maximum score of 9 points on the baseline test and 29 points on the retention test, respectively. An alpha of 0.74 was obtained for the retention test indicating acceptable internal consistency (see Table 3 in the Appendix for a full summary on test psychometrics).

### Sensitivity Analyses

Sensitivity analyses were conducted to investigate possible influences of the excluded data on the main results. Firstly, including participants that violated the study protocol in our analyses did not result in any significant differences versus the current reported results. Secondly, the mean difference in total retention test scores between groups was higher when the aberrant item of the baseline and retention test was not excluded, but remained not significant (*F*(1, 104) = 2.199, *p* = 0.141). However, the inclusion of this data significantly influenced the difference in retention test scores regarding the subset of original baseline test items (*F*(1, 104) = 3.956, *p* = 0.049).

### Narrative Comments

Generally, students who followed the spaced lecture responded positively towards the spaced lecture format. After the retention test, one student noted: “I really hope that we [the spaced lecture group], did better on the test, as I would really like to do this more often.” Several participants of the spaced lecture mentioned a positive effect of the intervening gaps on their attention and productivity. For example, a student said: “During those breaks I was totally distracted from the lecture material, resulting in a feeling of total reset. I really liked this as it enhanced my attention during the whole session.” Others were more doubtful: “I am not sure if the breaks improved my attention because I had a hard time to reboot after each break, therefore missing most of the small summaries.” The origami task was emphasized as an enjoyable distractor activity. “Normally, everyone grabs for his or her smartphone during occasions [breaks] like this . Now, everyone started the break trying to complete the origami task, and only switched to their phones when they gave up or failed.” Another student marked the negative side effect of this particular task: “ … at sudden moments I was too busy on thinking of what the next origami model would be than on the actual lecture content”*.* Lastly, there were some comments on the intensity of the spaced lecture: “I found it rather intense, I would hate to think of doing this four times in a row, but I could imagine it being preferable for a revision lecture.”

Students who followed the traditional lecture had some positive comments on the structure of the lecture but they generally agreed that they did not notice any difference with a normal lecture. On the question: “did you experience any differences in the lecture apart from starting and ending with the origami task?” one student answered: “hmm … well, to be honest, no.”

The lecturer enjoyed the spaced lecture format as she could recover during the breaks and experienced less fatigue afterwards. She noted: “I really had the idea that students’ attention on the last part [of the lectures] was higher than normal.”

## Discussion

This study investigated the effect of spaced learning during a lecture on medical students’ knowledge retention. We hypothesized that incorporating short spaces in the lecture would increase its effectiveness. We used an experimental design, comparing a spaced lecture with a traditional massed lecture. Our results showed that the effect of both lecture formats on knowledge retention was not significantly different. Notably, the positive narrative comments indicated that the spaced lecture format was generally well-received by students.

In our study, we incorporated short 5-min gaps between instruction sessions into a lecture to enhance the memory formation process. However, beneficial effects on knowledge retention were not found, suggesting that 5-min spaces might have been too short to stimulate the consolidation process. They may have been insufficient to overcome the refractory period needed for stabilization of the memory trace, for example. Apparently, 10-min spaces seemed to be more effective as Kelley and Whatson [24] were successful with their spaced learning strategy in the classroom where they incorporated short 10-min gaps. However, one should be careful interpreting these results, since findings can be highly dependent on the study design. For instance, in our study, we measured knowledge retention at 8 days, whereas Kelley and Watson measured it at 5 days. The 8-day period was selected since the Ebbinghaus forgetting curve indicates that forgetting declines exponentially and most of the forgetting occurs in the first week after initial learning [38, 39]. It might be that a shorter retention period, i.e., less than 8 days, had raised the ability to reveal differences between the study groups. This is in line with evidence indicating that short intervening gaps potentially promote advantages on shorter retention periods [8, 10, 41]. Another notable difference is that we chose to incorporate small summaries of preceding information into our lectures, whereas Kelley and Whatson repeated their 15-min instructional blocks three times. Our rationale for this design was that it was closer to the traditional teaching style and was expected to be an easy-to-incorporate tool for medical educators if it was found to be effective.

Despite some empirical evidence including our own study, researchers acknowledge that optimal spacing protocols for humans remain unknown and we may need to gain more fundamental knowledge of the mechanisms of memory formation in order to develop these protocols [23]. Our study contributes by informing the research community that 5-min spaces in a lecture setting seem insufficient to promote knowledge retention and that other approaches should be investigated to develop optimal spacing formats. In future studies on spaced learning during instruction, one may specifically investigate the influence of (1) the duration of spaces and (2) the number of spaces (3) in relation to the duration of the retention gap. Furthermore, one should be specific about the characteristics of the setting in which the study was performed, to determine if findings can be generalizable across educational contexts. Finally, future research may combine spaced learning with other effective learning strategies such as retrieval practice and/or test-enhanced learning to further promote knowledge retention in medical education [42–44].

### Limitations and Strengths

Our experiment was embedded in an obligatory course of the medical curriculum, so some practical limitations should be noted. For the sake of time and anonymity, we did not register attendees at the time of lecture. Consequently, we were unable to question students who were absent on the retention test about their reasons for a no-show. The higher dropout rate in the control group thus remains unexplained. This may also limit the impact of our results, as we do not know in which way these dropouts might have influenced the primary outcome. Additionally, we cannot be sure that students honestly indicated whether they had violated the study protocol. However, we assume a limited social desirability effect as students were informed that their participation had no effect on their course grade. Another limitation is that some sort of testing effect is inherent to our pre- post-test design. We aimed to minimize the testing effect by incorporating a 2-week gap between the baseline test and the intervention, and by including new questions in our retention test. Lastly, this study comprises one experiment in one session. Future research is needed that may focus on repeating and optimizing the spacing format to fully explore the potential of spaced learning and the influence of contextual factors.

Specific strengths of the study design should also be delineated. First, the protocol included reliable tests in which we did not observe any floor or ceiling effects. We showed an increase in overall test scores from 10 to over ~ 30%. However, it is hard to contextualize this result as previous literature on retention following a lecture is heterogenous, with a high variability of the moment of delayed testing, e.g., 1 week [45], 4 months [46], 5 months [47], type of testing, and restudying opportunities, e.g., summarize, note-taking, or self-questioning [48]. Second, the analyses included narrative comments which indicated that our new spaced lecture format was well-received. The majority of students and the lecturer noted that this format increased their attention and engagement and improved their productivity. It would be interesting to investigate whether this experience of enhanced attention could be quantified, using any approach that previously assessed mind wandering and its effect on retention in a lecture context [49, 50].

## Conclusion

Our findings showed that a spaced lecture did not enhance knowledge retention in medical students compared with a traditional, massed lecture. However, positive narrative comments indicated that this new spaced lecture format was generally well-received by students and the lecturer. Additionally, a theoretical and practical elaboration on our findings resulted in recommendations for educators and researchers on how to implement and study future spaced learning projects.

## Appendix

This table gives an overview of the retention test test statistics. For each question, a *p* value and Rir value were calculated. A *p* value indicates the difficulty level for a specific question and is equal to the proportion of participants that answered the question correctly. Consequently, a *p* value is expressed as a number between 0 and 1, and the more difficult the question was, the lower the score. The Rir value is an indicator of the distinctiveness of a question between better and worse performing students. In other words, it expresses the correlation between the performance on this question and total performance. Higher scores (max, 1) mean that if a certain question was answered correctly, this effectively differentiates between overall well and less well-performing students, negative values indicate an inverse correlation (the best students perform worse on this question).

**Table 3:** Retention test test analyses

	**Mean**	**SD**
Total score (points)	9.55	3.82
Total score (%)	32.94	13.18
KR-20 alpha	0.74	
**Question no.**	***p*****value**	**Rir**
Question 1	0.00	0.00
Question 2	0.11	0.46
Question 3	0.65	0.20
Question 4	*NA*	*NA*
Question 5	0.88	0.33
Question 6	0.88	0.17
Question 7	0.61	0.27
Question 8	0.34	0.38
Question 9	0.04	0.16
Question 10	0.07	0.07
Question 11	0.05	0.36
Question 12	0.88	0.22
Question 13	0.28	0.36
Question 14	0.03	0.27
Question 15	0.06	0.11
Question 16	0.36	0.25
Question 17	0.19	0.25
Question 18	0.66	0.27
Question 19	0.52	0.39
Question 20	0.28	0.28
Question 21	0.11	0.40
Question 22	0.05	0.32
Question 23	0.89	0.34
Question 24	0.14	0.24
Question 25	0.36	0.27
Question 26	0.43	0.33
Question 27	0.22	0.07
Question 28	0.04	-0.09
Question 29	0.50	0.30
Question 30	0.25	0.23
